# Inequalities in basic activities of daily living among older adults: ELSI-Brazil, 2015

**DOI:** 10.11606/S1518-8787.2018052000617

**Published:** 2018-10-25

**Authors:** Fabíola Bof de Andrade, Yeda Aparecida de Oliveira Duarte, Paulo Roberto Borges de Souza, Juliana Lustosa Torres, Maria Fernanda Lima-Costa, Flavia Cristina Drumond Andrade

**Affiliations:** I Fundação Oswaldo Cruz. Instituto René Rachou. Núcleo de Estudos em Saúde Pública e Envelhecimento. Belo Horizonte, MG, Brasil; II Fundação Oswaldo Cruz. Instituto René Rachou. Programa de Pós-Graduação em Saúde Coletiva. Belo Horizonte, MG, Brasil; III Universidade de São Paulo. Escola de Enfermagem. São Paulo, SP, Brasil; IV Fundação Oswaldo Cruz. Instituto de Comunicação e Informação Científica e Tecnológica em Saúde. Rio de Janeiro, RJ, Brasil; V University of Illinois at Urbana-Champaign. Kinesiology and Community Health. Champaign, IL, USA

**Keywords:** Aged, Activities of Daily Living, Disabled Persons, Socioeconomic Factors, Health Inequalities

## Abstract

**OBJECTIVE:**

To evaluate the magnitude of wealth-related inequalities in basic activities of daily living among community-dwelling Brazilian older adults and to determine the contribution of demographic, socioeconomic, and health conditions to the inequality.

**METHODS:**

We used data from the 2015 Brazilian Longitudinal Study of Aging (ELSI-Brazil) with a nationally representative sample of adults aged 50 years or older. We assessed wealth-related inequalities in basic activities of daily living by the concentration index. Concentration index was decomposed to determine the contribution of demographic, health, and socioeconomic factors to wealth-related inequalities in basic activities of daily living.

**RESULTS:**

The prevalence of disability in the sample was 15.7% (95%CI 14.9–17.6). The concentration index was -0.145 (95%CI -0.194– -0.097), which indicates that disability is concentrated in the poorest individuals in Brazil. Inequalities in basic activities of daily living disability are primarily explained by socioeconomic status (wealth and own education) not by demographic or health factors.

**CONCLUSIONS:**

There are avoidable wealth-related inequities for those with a disability in Brazil. The strong contribution of the socioeconomic status highlights the need for new public health policies that promote equity, universality, and integrality, in addition to the expansion of home nursing public services.

## INTRODUCTION

Brazil is among the largest countries in the world and the aging process of its population is occurring at a very fast pace^1^. With a higher proportion of individuals reaching older ages, disability becomes a more prevalent health problem. According to the Brazilian National Health Survey conducted in 2013, 30.1% of the Brazilian older adults (60 years or older) reported having difficulty performing basic activities of daily living (BADL), which are related to survival activities such as eating and dressing, or performing instrumental activities of daily living (IADL), which are related to community activities such as managing money and taking medications. Moreover, prevalence levels increase dramatically with age – 16.4% among the older adults aged 60–64 years reported having ADL disability compared to 48.3% among those aged 75 and older^2^. Although most persons will experience health declines in later life, disability is not equally distributed across social groups and individuals from higher socioeconomic status (SES) often experience better functional health^3^ ^,^ ^4^.

Health disparities by region, education, income, wealth, and social background are evident in Brazil among adults^5–7^ and older adults^3,4,8–10^. These disparities emerge despite the fact that Brazil has a national health system [*Sistema Único de Saúde* (SUS)] guided by the principle of social equity. Despite expansions in the SUS, there is evidence that health inequalities related to disability have increased among Brazilian older adults from 1998 to 2008^8^. Among older adults aged 60–64 years, these inequalities almost doubled during this period^8^.

Previous research has found social inequalities for disability among older adults in Brazil^3,4,7–9^, particularly across educational groups^3^ ^,^ ^4^, household income^4^, and wealth^3^. Even though these studies have been critical in identifying demographic, socioeconomic, and health determinants of disability, the contribution of these determinants to the socioeconomic inequalities in disability need to be better understood. Understanding which factors are stronger contributors to inequalities is critical to advance policies as some factors are amenable to these policies, such as education, but others, such age and sex, are not.

In this study, we focus on the concentration index (CI) to describe the magnitude of inequality. The CI is now widely used in studies focusing on socioeconomic inequalities in health around the world, but it has only just now started being used in Brazil. The CI has the advantage of describing the socioeconomic inequality using the entire socioeconomic distribution^11^ rather than focusing on comparisons between extreme groups (e.g. highest *versus* lowest wealth quintile). We believe that using the entire distribution is a better way to assess the social gradients in disability^12^. In addition, previous studies have not attempted to decompose the determinants of health inequalities. This study fills this gap and estimates the magnitude of wealth-related inequalities in BADL among community-dwelling Brazilian older adults using the CI. In addition, it uses decomposition analyses to determine the contribution of demographic, socioeconomic, and health determinants to the overall inequality.

## METHODS

### Data Source

This study used data from the baseline of the Brazilian Longitudinal Study of Aging (ELSI-Brazil), which is a nationally representative, population-based cohort study of persons aged 50 years or older conducted between 2015 and 2016.

The ELSI-Brazil used a probabilistic sample and applied a sampling procedure that combined geographical stratification and clustering in three stages. The municipalities were the primary sample units, followed by census tracts and households. All residents in the selected households aged 50 years or older were eligible for interview. The final sample comprised 10,000 older adults (9,412 participated) residing in 70 municipalities from different Brazilian regions. Further details of the ELSI-Brazil can be seen elsewhere^a^ ^,^ ^13^.

The 2015 ELSI-Brazil followed the standards set by the Declaration of Helsinki and was approved by the ethics board of the Oswaldo Cruz Foundation, Minas Gerais (CAAE 34649814.3.0000.5091). All participants signed an informed consent form.

### Measures

Self-reported disability in six BADL measures – walking across a room, dressing, bathing, eating, getting in and out of a bed (transferring), and toileting – was used to measure disability. First, we dichotomized each BADL measure with a score of “0” indicating that the respondent did not have any disability and a score of “1” indicating that the respondent had any difficulty (little, great, unable) to perform the activity. Next, we created a dichotomous variable in which a score of “0” was given to the individuals who did not have any limitations and a score of “1” was assigned to those who reported having difficulty performing at least one of the six BADL measures.

The wealth index was used to describe the socioeconomic status of the older adults. The index was constructed using principal component analysis with information on household ownership of durable goods and housing characteristics according to the following variables: household assets (internet, television, cable tv, refrigerator, washing machine, dishwasher, clothes dryer, computer, desk phone, cell phone, microwave, motorcycle, car) and household characteristics (maid, wall of masonry or wood, piped water, paved street, bathroom). The wealth index was categorized into quintiles.

Determinants of disability were classified into the groups of demographic, health, and socioeconomic determinants. Demographic determinants included age (50–59, 60–69, and 70 years or older) and sex (female or male). Health determinants included the number of chronic conditions. Health conditions included self-reported hypertension, diabetes, cardiovascular disease, chronic obstructive pulmonary disease, arthritis, stroke, asthma, cancer, and renal insufficiency. Individuals were categorized as having no or one chronic condition or having two or more. Socioeconomic determinants included wealth, own education (0–3, 4–7, 8–11, and 12 or more years of schooling), and parental education (no formal education; incomplete primary school; complete primary school or more).

### Statistical Methods

Descriptive statistics were calculated for the sample. Given the complex survey design, associations between categorical variables and disability were assessed using the Rao-Scott chi-square test, which is a design-adjusted version of a Pearson chi-square test. Statistical inferences were considered significant at the p < 0.05 level.

We then estimated the CI to quantify the degree of wealth-related inequality in disability. The CI provides a summary measure of the relative inequality in disability that uses the entire socioeconomic distribution rather than just comparing the extremes^11^ of the distribution, such as the difference in prevalence between the highest and lowest wealth quintiles. The CI is based on the concentration curve that plots the cumulative percentage of the population ranked by wealth quintiles on the horizontal axis and the cumulative percentage of disability on the vertical axis. If there is no wealth-related inequality, CI will be zero and the concentration curve will be along the 45^o^ line (equality line). When the concentration curve lies below the equality line, the CI is positive, which indicates a disproportionate concentration of disability among the wealthier. When the curve is above the equality line, the CI is negative, which indicates a disproportionate concentration of disability among poor individuals^12,14–16^. The CI is bound between -1 and 1. We decomposed the CI to assess the contribution of each determinant to wealth-related inequality. The contribution of each determinant depends on the sensitivity (elasticity) of disability with respect to that factor and the degree of wealth-related inequality associated with that factor. The greater the sensitivity and the more unequal the factor is distributed in relation to wealth, the larger the contribution of that factor.

The horizontal inequity index (HI) was used to assess whether individuals with similar demographic and health characteristics have the same levels of disability irrespective of their socioeconomic status^12^. The HI is a measure of health inequity and was estimated based on the difference between CI and the contribution to the concentration index (CCI) from demographic and health characteristics. A positive HI means that there are inequities favoring the wealthier older adults, whereas a negative HI means that inequities are more common among poor older adults.

All analyses accounted for the complex survey design and included survey weights. Descriptive analyses were carried out using Stata 13 SE (Stata-Corp., College Station, Texas, USA), and the software ADePT was used for estimating the HI and the CI and its decomposition^17^.

## RESULTS

The prevalence of disability in the sample was 15.7% (95%CI 14.9–17.6). The characteristics of the participants and results of bivariate analyses are presented in Table 1. Most of the participants are aged 50–59 years (48.4%) and male (53.5%) and they have no or one chronic condition (63.9%). Age, education (own and parental), wealth, and chronic conditions are statisticaly associated with BADL disability. The Figure shows the proportion of the participants with BADL disabilities by age group and sex. Differences across age groups were statistically significant with disability increasing with age, particularly after age 70 (p < 0.0001). Prevalence of BADL did not differ by sex.

**Table 1:** Descriptive and bivariate analysis by disability status. Brazilian Longitudinal Study of Aging (ELSI-Brazil), 2015–2016.

Variable	Total	No BADL	BADL	p
Unweighted sample size	8,642	7,175	1,467	
Age				
50–59	48.4	50.0	39.8	< 0.0001
60–69	29.6	30.2	26.7	
≥ 70	21.9	19.8	33.5	
Sex				0.1120
Female	46.5	47.0	43.6	
Male	53.5	53.0	56.4	
Own education (years of schooling)				< 0.0001
0–3	32.3	29.9	45.2	
4–7	31.1	31.0	31.7	
8–11	28.0	29.5	19.7	
≥ 12	8.7	9.6	3.4	
Parental education				< 0.0001
No formal education	49.8	48.2	58.6	
Incomplete primary school	22.2	22.4	21.6	
Complete primary school or more	28.0	29.5	19.7	
Wealth quintile				< 0.0001
1 (poorest)	19.8	18.9	24.2	
2	20.0	19.0	25.1	
3	19.7	19.4	21.3	
4	20.3	20.7	18.2	
5 (wealthiest)	20.3	22.0	11.3	
Number of health conditions				< 0.0001
0–1	63.9	68.2	41.1	
≥ 2	36.1	31.8	58.9	

BADL: basic activities of daily living – walking across a room, dressing, bathing, eating, getting in and out of a bed (transferring), and toileting

**Figure:**
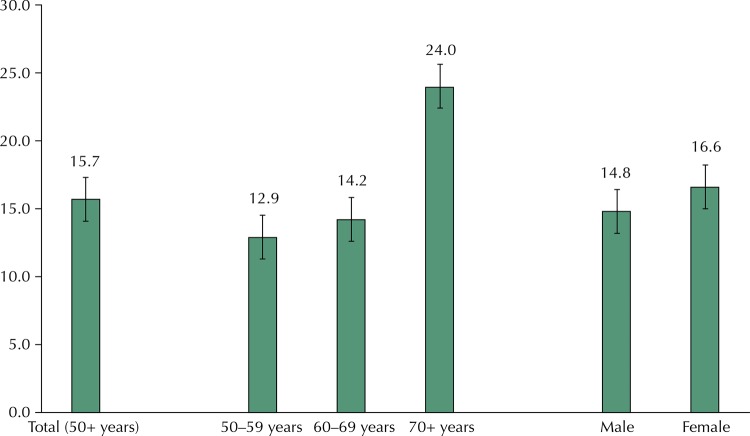
Proportion of BADL disability according to age group and sex. Brazilian Longitudinal Study of Aging (ELSI-Brazil), 2015–2016.

BADL: basic activities of daily living

Table 2 shows the values of CI and HI and the contributions to the concentration index (CCI) based on the decomposition analyses. The negative value for CI (-0.145, 95%CI -0.194– -0.097) demonstrates that BADL is concentrated among poor older adults in Brazil. In addition, the negative value for HI (HI = -0.143, 95%CI -0.189– -0.097) highlights that poor older adults are more likely to have BADL even after accounting for differences in demographic and health determinants. Older age, female sex, and lower education have negative CI, which means that these determinants are more concentrated among the poor. In contrast, having two or more health conditions and higher parental and own education have positive CI, which implies that these determinants are more concentrated among the wealthier. Overall, the results indicate that the aggregate contribution of demographic and health factors was only 1.8%, whereas socioeconomic factors, particularly wealth and own education, were the main contributors (92.2%) to the inequality in BADL disability.

**Table 2:** Estimates of the concentration index (CI), horizontal index (HI), and decomposition of the concentration index. Brazilian Longitudinal Study of Aging (ELSI-Brazil), 2015–2016.

Variable	Disability		

	CCI	%	CI
Age (years)			
50–59 (reference)			
60–69	0.000	-0.1	-0.005
≥ 70	-0.006	4.2	-0.095
Female	0.000	-0.3	-0.032

(1) Total demographic factors	-0.006	3.8	

Number of health conditions			
0–1 (reference)			
≥ 2	0.003	-2.0	0.010

(2) Total health factors	0.003	-2.0	

(3) Total demographic and health (1) + (2)	-0.003	1.8	

Wealth			
1 – poorest (reference)			
2	-0.002	1.4	-0.405
3	0.000	-0.1	-0.009
4	-0.010	7.1	0.391
5 – wealthiest	-0.064	43.9	0.797
Parental education			
No formal education (reference)			
Incomplete primary school	0.000	0.1	0.023
Complete primary school or more	-0.007	5.1	0.304
Own education (years)			
0–3 (reference)			
4–7	0.003	-1.9	-0.043
8–11	-0.026	17.9	0.272
≥ 12	-0.027	18.7	0.581

(4) Total SES	-0.134	92.2	

(5) Total (3) + (4)	-0.137	94.0	

(6) Error	-0.009	6.0	

(7) Total CI (5) + (6)		100	-0.145
95%CI			(-0.194– -0.097)

HI (7) - (3)			-0.143
95%CI			(-0.189– -0.097)

CCI: contribution to concentration index; %: percent of the concentration index explained by the variable; CI: concentration index; HI: horizontal inequality index considering sample weights; BADL: basic activities of daily living – walking across a room, dressing, bathing, eating, getting in and out of a bed (transferring), and toileting; SES: socioeconomic status

## DISCUSSION

Based on a nationally representative sample, this article shows that disabilities in activities of daily living affect approximately 16% of the Brazilian older adults. It also demonstrates the existence of wealth-related inequalities in BADL disabilities among adults aged 50 years or older in Brazil. To our knowledge, this is the first study to provide estimates of the CI and to decompose wealth-related inequalities in disability in Brazil. The study revealed that BADL disabilities are concentrated among poor older adults. Additionally, the inequalities in BADL disability were mainly explained by SES (wealth and own education), and a smaller percentage was explained by demographic and health factors. Analyses controlling for demographic and health factors highlighted the inequities in disability among older adults in Brazil. Even though the burden of disability is greater among poor older adults in Brazil, the Family Health Strategy, a government program aimed at improving access to health services, still lacks coverage for nearly 30% of the poorest individuals in Brazil^18^.

Socioeconomic inequalities in disabilities have been consistently reported in Brazilian studies^3–6,8,^ ^9^. These studies provide important information about the social determinants of disability in Brazil and, in general, they indicate that individuals with lower education and income have worse health indicators than those who are better off^3,4,7–9^. However, the comparison of our study with these previous studies is difficult given the different measures of inequality used, and they also have not provided the varying contributions of different factors to that inequality. Our study addressed some of the limitations in the existing literature and examined the contributions of demographic, health, and socioeconomic determinants to inequality.

The decomposition of the concentration index distinguished the contributions of each determinant related to wealth-related inequality in BADL. Some factors, such as age and sex, are not amenable to changes in policy, whereas socioeconomic conditions can respond to these changes. Identifying which determinant contributes to a larger share of wealth-related inequalities in disability is important as socioenomic inequalities are avoidable^14^ ^,^ ^19^. Despite the differences in the prevalence of disability by age and sex, we found that demographic factors explain only a small percentage of BADL disparities. In fact, SES explained most BADL inequalities. Wealth contributed to more than half of the total inequality, while own education contributed to nearly 35% of that total inequality. These findings reinforce the importance of examining the various determinants of health inequalities in old age, as each determinant can carry unique contributions^20^.

The fact that SES determinants explain most of the inequalities in disability highlights the need for changes in policies related to improving educational levels, reducing economic inequalities, and improving health care access. Evidence from longitudinal data shows that individuals with no schooling not only have higher rates of disability onset, but they also have lower rates of recovery^21^. Given that educational levels are typically established by early adulthood and are relatively constant among older persons^22^, investiments in education during early years are needed. Improving the economic conditions of individuals at the bottom of the social gradient can also improve disability inequalities. Persons economically worse off tend to have less opportunities to obtain higher education levels, because of factors such as workload, difficulty in paying for private school^6^, and overall lower prestige, status, and control^23^. Nonetheless, current socioeconomic circumstances matter as well^24^. Persons with lower SES may have a higher likelihood of a health condition resulting in activity limitation. Poor health conditions and disability may lead to additional expenditures (such as health care, transportation, assistive devices, personal assistance, and house adaptation)^6^ that can overwhealm families and individuals at the bottom of the social gradient. Persons who are wealthier in old age can afford a wider range of facilities to either improve or keep good physical functioning. Under these circumstances, a possible way to reduce inequalities in disability at older ages may be providing more equitable, universal, and better care to those who cannot afford it. There is some evidence that access to health care and doctor visits have been increasing in Brazil^25^, which in turn may be reducing the gap in health levels between SES groups. Therefore, expanding public health care and rehabilitation services^6^ and improving accessibility^26^ and home care are important ways to reduce the inequalities^3^.

Even though chronic conditions contributed to a small share of the inequality, they were more concentrated among the wealthiest individuals. However, information on chronic conditions is based on self-reported data, in which knowledge and reporting can differ across SES groups. Wealthier individuals are more likely to have a diagnosed chronic condition as they have more access to healthcare^27^. Often, healthier lifestyles such as engaging in physical activity are also concentrated among wealthier individuals. As a result, wealthier individuals can live longer with diseases and may be less likely to experience disability.

This study has important strengths. First, the findings of this study are based on data from a nationally representative sample of older adults in the largest country in Latin America. Second, the data also included several socioeconomic indicators that were used to examine their contribution to wealth-related inequalities in disability. Finally, to the best of our knowledge, this was the first study to measure wealth-related inequality using CI and to decompose the contribution of its determinants. Nonetheless, the study is based on cross-sectional data that prevents us from analyzing how changes in socioeconomic conditions influence disability trends. In addition, the socioeconomic ranking was derived from a measure of wealth that was based on an index of household assets and characteristics, which does not include other wealth domains, such as savings, that can be associated with living standards. Moreover, the data were collected from community-dwelling individuals, thus excluding the ones in institutions who are more likely to be disabled. This in turn has the potential to increase the prevalence of BADL disability. However, less than 1% of the older adults in Brazil are in long-term care institutions^28^.

This study showed that avoidable wealth-related inequities persist in BADL disability. The strong contribution of SES factors highlights the need for improvements in public health policies that promote equity, universality, and integrality, such as the Family Health Strategy, and improve home nursing public services. To increase the effectiveness of preventive and rehabilitation strategies, which reduce activity limitations, it is necessary to go beyond the determinants of disability, fostering health-related behaviors and promoting mental health.It is necessary to combine those actions with interventions focused on social determinants, particularly among the poorest individuals in the country.

## Footnotes

a Fundação Oswaldo Cruz. Brazilian Longitudinal Study of Aging. Rio de Janeiro; c2015 [cited 2017 Nov 28]. Available from: http://elsi.cpqrr.fiocruz.br
